# Temporomandibular Joint Regenerative Medicine

**DOI:** 10.3390/ijms19020446

**Published:** 2018-02-02

**Authors:** Xavier Van Bellinghen, Ysia Idoux-Gillet, Marion Pugliano, Marion Strub, Fabien Bornert, Francois Clauss, Pascale Schwinté, Laetitia Keller, Nadia Benkirane-Jessel, Sabine Kuchler-Bopp, Jean Christophe Lutz, Florence Fioretti

**Affiliations:** 1 INSERM (French National Institute of Health and Medical Research), UMR 1260, Regenerative Nanomedicine (RNM), FMTS, 11 rue Humann, 67000 Strasbourg, France; dr.xvb@laposte.net (X.V.B.); ysiaidouxgillet@free.fr (Y.I.-G.); marion.pugliano@gmail.com (M.P.); strub.marion@orange.fr (M.S.); bornertfabien@gmail.com (F.B.); francois_clauss@hotmail.com (F.C.); pschwinte@unistra.fr (P.S.); lkeller@unistra.fr (L.K.); nadia.jessel@inserm.fr (N.B.-J.); kuchler@unistra.fr (S.K.-B.); Jean-Christophe.LUTZ@chru-strasbourg.fr (J.C.L.); 2 Faculté de Chirurgie Dentaire, Université de Strasbourg, 8 rue Ste Elisabeth, 67000 Strasbourg, France; 3 Médecine et Chirurgie Bucco-Dentaires & Chirurgie Maxillo-Facial, Hôpitaux Universitaires de Strasbourg (HUS), 1 place de l’Hôpital, 67000 Strasbourg, France; 4 Faculté de Médecine, Université de Strasbourg, 11 rue Humann, 67000 Strasbourg, France

**Keywords:** temporomandibular joint, regenerative medicine, stem cells, scaffolds, growth factors, functionalization, drug delivery systems, nanotechnology, osteochondral regeneration

## Abstract

The temporomandibular joint (TMJ) is an articulation formed between the temporal bone and the mandibular condyle which is commonly affected. These affections are often so painful during fundamental oral activities that patients have lower quality of life. Limitations of therapeutics for severe TMJ diseases have led to increased interest in regenerative strategies combining stem cells, implantable scaffolds and well-targeting bioactive molecules. To succeed in functional and structural regeneration of TMJ is very challenging. Innovative strategies and biomaterials are absolutely crucial because TMJ can be considered as one of the most difficult tissues to regenerate due to its limited healing capacity, its unique histological and structural properties and the necessity for long-term prevention of its ossified or fibrous adhesions. The ideal approach for TMJ regeneration is a unique scaffold functionalized with an osteochondral molecular gradient containing a single stem cell population able to undergo osteogenic and chondrogenic differentiation such as BMSCs, ADSCs or DPSCs. The key for this complex regeneration is the functionalization with active molecules such as IGF-1, TGF-β1 or bFGF. This regeneration can be optimized by nano/micro-assisted functionalization and by spatiotemporal drug delivery systems orchestrating the 3D formation of TMJ tissues.

## 1. Introduction

### 1.1. Temporomandibular Joint (TMJ)

The temporomandibular joint (TMJ) is an articulation covered by dense fibrocartilage formed between the mandibular condyle and the temporal bone. The temporal articular surface is large and consists of the mandibular fossa and the articular tubercle. Along this large articular temporal surface, each mandibular condyle has a wide motion range, consisting of both rotation and translation.

Fibrocartilaginous disc cushions mechanical stresses that exist between the temporal and mandibular articular surfaces. The high collagen content of this disc provides great rigidity and durability. The TMJ disc has no direct vascularization or innervation by itself. However, its posterior attachment, known as retrodiscal tissue, features many vessels and nerves which are crucial during physio-pathological processes.

### 1.2. Temporomandibular Joint Disorders (TMJD)

TMJD (temporomandibular joint disorders) are very common, their prevalence being around 52% [1]. TMJD is a general term actually covering a large number of clinical occurrences affecting the TMJ and masticatory-related structures. They cover in various etiologies: traumatic, inflammatory, and congenital. TMJD are also characterized by deficient wound healing and fibrosis caused by continuous and irreversible injuries.

Pain, malocclusion, limited range of motion, deviation, joint clicking and clenching are most of time associated with TMJD. These disorders are often so painful during basic oral activities (eating and speaking) that quality of life of patients is greatly impacted [2]. Pain is the primary symptom and the main reason why patients are referred to practitioners to seek treatment.

**Figure 1:**
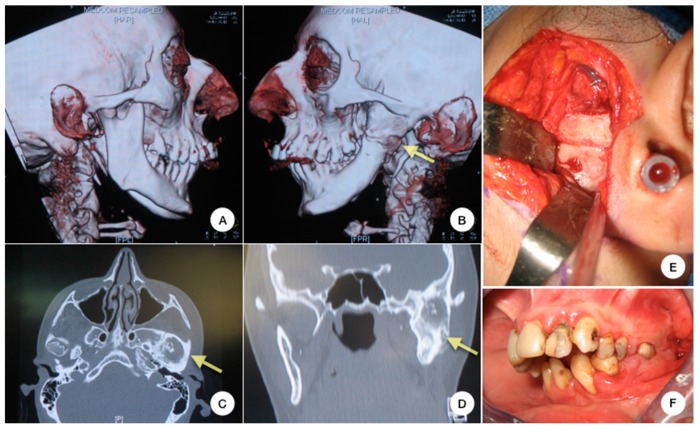
Invasive treatment of a patient suffering from TMJ (temporomandibular joint) ankylosis: Right lateral view of a 3D CT scan reconstruction of the head: the right TMJ is affected by joint space narrowing (**A**) and the left TMJ space has completely disappeared and been replaced by an osseous block (**B**). This replacement of the left TMJ by an osseous block of ankylosis is seen on transversal (**C**), and on coronal (**D**) CT scan sections. Intraoperative view of invasive treatment: the osseous bloc of ankylosis replacing the left TMJ space is approached through a pre-auricular incision (**E**). Preoperative intraoral photograph showing the absence of mouth opening (**F**).

Osteoarthritis-like degenerative joint disease belonging to TMJD is a destruction of bone and cartilage with a consecutive inflammation which enhances tissue destruction [3]. TMJ degeneration features are: displacement, thickening and/or disc perforation, whole destruction of articular fibrocartilage and crucial modifications of bone remodeling such as sclerosis or periarticular osteophyte formation [4,5]. The ultimate stage of degeneration can even result in the replacement of TMJ by a block of fibrous and bony tissue, namely, ankylosis [6] (Figure 1).

## 2. Current Status of Temporomandibular Treatments

### 2.1. Current Therapies

Once the primary factors of TMJD have been identified and eliminated, the treatment can vary according to the severity of the disorder: non-invasive, minimally invasive and invasive procedures. However, permanent recovery is rarely obtained and therefore, follow-up therapies are required [7].

Occlusal orthodontics, medications, physical therapy and acupuncture are the most common non-invasive treatments. Although occlusal orthodontics are widely used, their effectiveness remains questionable [8,9]. Non-invasive medications consist anxiolytics, muscle relaxants, non-steroidal anti-inflammatory drugs and opioids [4,10]. Similarly, there is no consensus regarding the long-term effectiveness of such oral or topical medications [11].

Minimally invasive treatments can target either extrinsic masticatory muscles or TMJ itself. Masticatory muscles (masseter, temporalis and lateral pterygoid) have been injected with botulinum toxin type A (Botox) for thirty years [12,13]. The findings of a literature review conducted using The Cochrane Controlled Trials Register between 1980 and 2012 suggest that there is level 1 evidence supporting the efficacy of Botox in the treatment of TMJD [14].

Minimally invasive treatments of TMJ itself include intra-articular injections, arthrocentesis, and arthroscopy. Intra-articular injections of corticosteroids into either one or both joint compartments improve TMJD symptoms [15,16]. Arthrocentesis is a lavage of the superior TMJ space by a saline solution. The pressure generated by irrigation may remove adhesions. Some authors proposed morphine irrigation after arthrocentesis [17]. The short and long term efficacy of this minimally invasive treatment is well-documented [17,18,19]. The miniaturization of endoscopes allowed TMJ arthroscopy. It provides adequate visualization and manipulation of pathological intra-articular tissues [20]. Small-diameter instruments permit their direct removal with a high rate of success [20,21].

Invasive treatment is the only option for patients suffering from ankylosis, neoplasia, dislocation, and developmental disorders [22,23]. It consists of open joint surgery, namely arthrotomy, which aims at either restoring joint tissues or completely replacing the TMJ with autogenous or alloplastic material (Figure 1).

Regarding invasive treatment of the TMJ disc, both its surgical repositioning and the removal of affected tissues have short-term efficacy [24]. The surgical removal of the entire affected TMJ disc (discectomy) is most of the time prescribed [25]. Although discectomy has a significant long-term efficacy, it does not prevent from osteoarthrosis [26]. Along with poor remodeling of the mandibular condyle, destruction of its articular surface or osteophyte formation can occur [26]. It has been shown in vivo also that a functional disc is crucial for mandibular condyle regeneration [27].

The many options proposed for disc replacement have not shown satisfactory clinical outcomes: teflon-proplast or silicone synthetic implants [28,29], sear cartilage, autologous dermal graft [26], full-thickness skin graft, or pedicled temporalis muscle flap [23,30,31]. For instance, the clinically tested TMJ disc implants did not provide any significant advantage over discectomy, except for the reduction of clicking [4].

For severe TMJ degeneration, the total surgical joint replacement is currently prescribed. The first experiments on regeneration using alloplastic TMJ implants showed a tendency for premature failure. Subsequent immune reactions were triggered, therefore resulting in catastrophic joint damage [32,33]. Thus, TMJ regeneration strategies have been reconsidered with more caution. At the moment, the consensual strategy seems to recommend reconstruction of the TMJ using autologous tissues for young patients, and TMJ replacement using metallic prostheses for adults. With conventional alloplastic strategies, the articular surfaces can be affected by erosion or heterotopic bone formation [34]. Therefore, two-piece 3D custom-made prostheses include an ultra-high molecular weight polyethylene (UHMWPE) implant for the replacement of the temporal articular surface (mandibular fossa). Such prostheses seem to currently be the most satisfying solution available for total surgical joint replacement [35,36].

Tissue engineering of TMJ has been a primary concern in scientific discussion and practice. The limitations of current therapeutics for TMJD have led to an increased interest in regenerative strategies combining cells, implantable scaffolds and well-targeting bioactive molecules. The recent advances of regenerative medicine for orthopedics may contribute to meet the challenge of this specific complex regeneration.

### 2.2. Challenging Regeneration

Meeting the demands for anatomic, structural, and functional regeneration of TMJ is very challenging. Innovative strategies and biomaterials are absolutely crucial because TMJ can be considered as one of the most difficult tissues to regenerate.

The TMJ is an anatomic zone so narrow and dense that surgical access is not easy. Its limited blood supply and hence its very limited healing capacity do not facilitate regenerative strategies either.

The mechanical and structural (three-dimensional) properties of the mandibular condyle implant must match that of anatomic condyle for human clinical applications. Also, the implanted tissue-engineered mandibular condyle must have rapid and adequate remodeling potential allowing oral functions.

**Figure 2:**
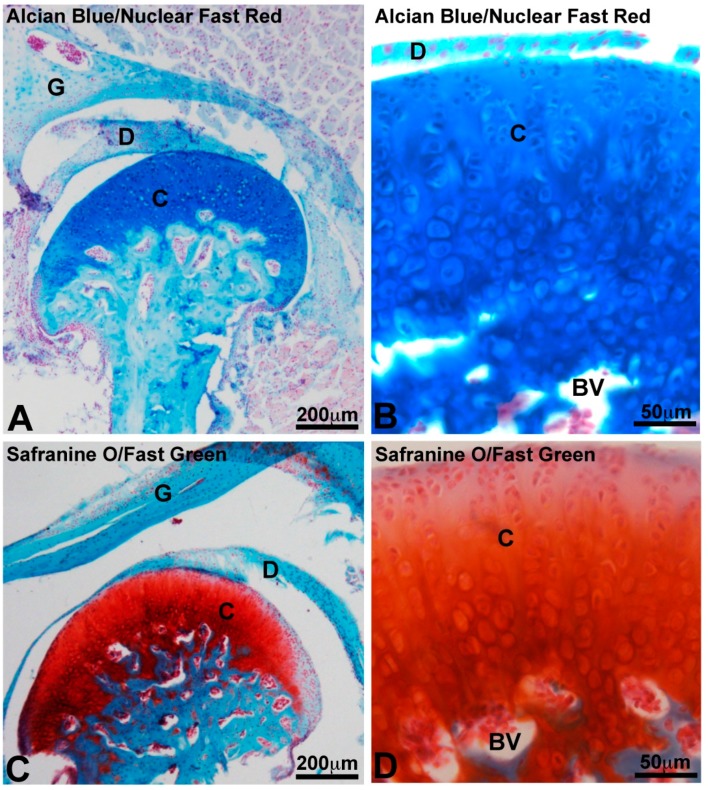
Histological organization of murine TMJ. TMJ is an articulation between the glenoid fossa of temporal bone (G) and mandibular condyle (C). TMJ disc cushioning articular mechanical stresses is fibrocartilaginous (D). TMJ condyle is made of a specific articular cartilage and a underlying bone containing blood vessels in medullar spaces (BV). Alcian Blue/Nuclear Fast Red specifically staining of mucopolysaccharides in blue (**A**,**B**) and Safranine O/Fast Green staining of cartilaginous proteoglycans in orange/red (**C**,**D**) highlight the osteochondral interface.

Histologically, the TMJ cartilage differs a lot from the other hyaline articular cartilages. Thus, regenerating this unique articular cartilage with its complex structure and organization is very tricky. TMJ regeneration means to be able to engineer a mandibular condyle implant with its unique cartilage and its underlying bone in a single osteochondral construct. The challenge in TMJ regeneration is to promote matrix synthesis and tissue maturation of chondrogenic and osteogenic cells in suitable scaffolds containing active molecules which are able to separately orchestrate osteogenesis and chondrogenesis (Figure 2).

The success of TMJ regenerative strategy is not only measured by the restoration of function but also by the long-term prevention of ossified or fibrous adhesions which are the main complications of engineered TMJ replacements [37]. Thus, pro-regenerative active molecules incorporated in scaffolds of engineered TMJ must also prevent any ossifications and any adhesions.

## 3. Histology and Macromolecular Biology of the TMJ

**Figure 3:**
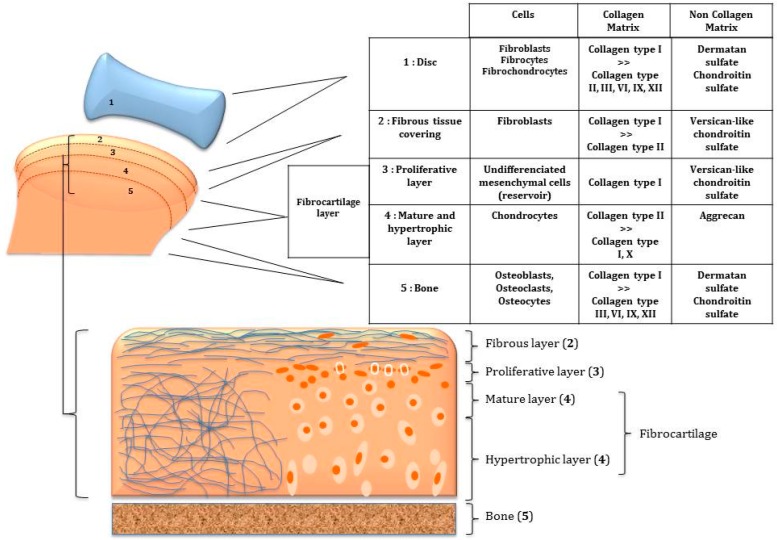
Scheme of the composition of the five compartments of TMJ to regenerate. Their cellular and macromolecular compositions differ of lot. An osteochondral molecular gradient of functionalization able to orchestrate the 3D formation of different TMJ tissues is the key of its regeneration.

**Figure 4:**
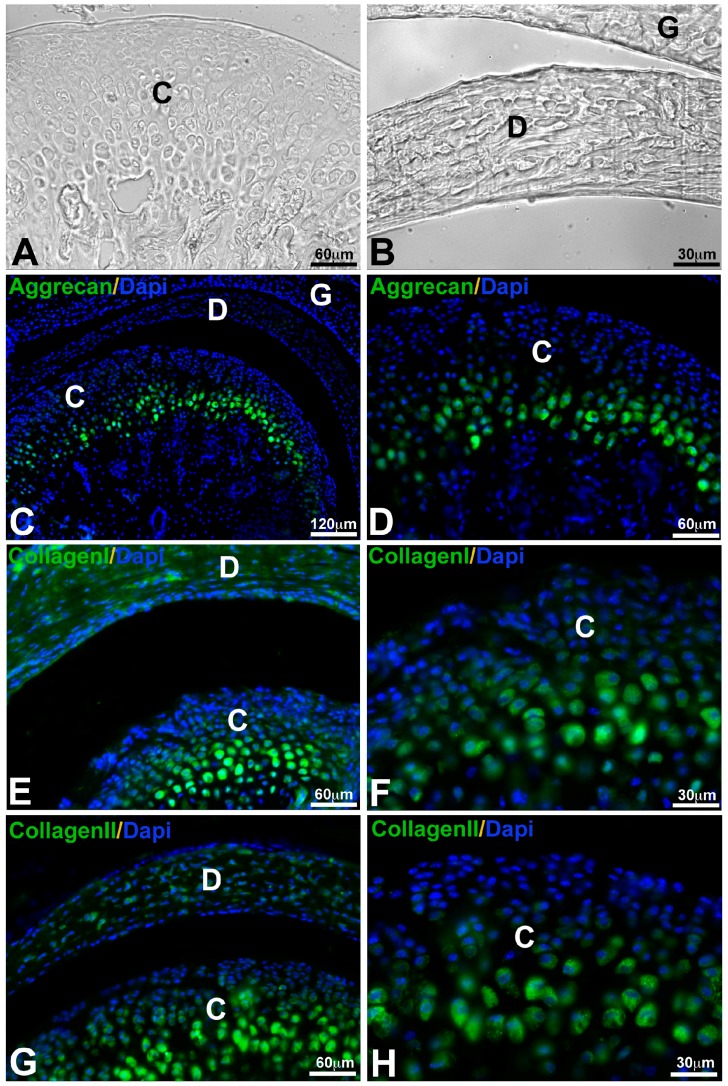
Expression of different conjunctive macromolecules in murine TMJ detected by immunofluorescence. TMJ observed by phase contrast microscope (**A**,**B**), Aggrecan expressed by chondrocytes in hypertrophic layer of mandibular condyle (**C**,**D**), type I collagen in the disc and in the fibrocartilage layer of mandibular condyle (**E**,**F**) and type II collagen in the fibrocartilage layer of mandibular condyle (**G**,**H**). Nuclei were stained with 4′,6-diamidino-2-phenylindole (DAPI). Condyle (**C**); Disc (**D**); Glenoid fossa (**G**).

There is a continuous debate about the embryonic origin of mandibular articulating surface: blastemal or periosteal origin [38,39]. The thickness of condylar fibrocartilage in humans can reach 0.48 mm as a maximum and is subject to variations caused by age and functional conditions [40,41]. The articular surface of most synovial joints is covered by hyaline cartilage. It is not the case of the TMJ which has an articular surface covered by a layer of fibrous tissue [42]. This fibrous zone contains abundant type I collagen, while collagen type II is minimally present. Underlying this superficial fibrous zone, a fibrocartilage layer is described which can be subdivided schematically into proliferative and hypertrophic zones [42,43]. The proliferative zone functioning as a cell reservoir [44] is rich in type I collagen. In the fibrous and proliferative zones, the orientation of collagen fibers was revealed as anisotropic with initiation of antero-posterior alignment of the fibers [43,45]. A chondroitin sulfate-based proteoglycan resembling versican predominates in both of these zones [46,47]. The hypertrophic zone is rich in chondrocytes, in aggrecan and in collagen type II. Collagen types I and X are also detected [47]. TMJ condylar fibrocartilage contains less glycosaminoglycans (GAGs) than hyaline articular cartilage [48] (Figure 3 and Figure 4).

The TMJ disc attached to the condyle and temporal bone by fibrous connective tissue measures 14 mm antero-posteriorly and 23 mm medico-laterally in humans [49]. Its periphery is thicker than the center, consequently its shape is biconcave. Populations of cells found in the TMJ disc differ from those of hyaline cartilage and are heterogeneous: fibroblasts, fibrocytes and fibrochondrocytes [50]. The periphery and attachments of the disc are well-vascularized but its central heart is avascular [51]. Type I collagen predominates but other collagens are found: types II, III, VI, IX, and XII [52]. Collagens in the disc are mostly anisotropic [53]. Orientation of fibers is antero-posterior in the center and more circumferential in the peripheral area [53]. Human disc mechanical tensile properties in anteroposterior and mediolateral directions reflect this anisotropy of the collagen fiber arrangement [49]. The particularity of these collagen fibers is to be crimped. It may improve the mechanical properties of the disc, in particular under tension [54]. TMJ disc contains less glycosaminoglycans (GAGS) than hyaline articular cartilage [55]: fraction of GAGS ranges from 1 to 10% by dry weight. Dermatan sulfate and chondroitin sulfate are the most abundant GAGS [56] (Figure 3 and Figure 4).

## 4. TMJ Tissue Engineering

### 4.1. Cell Strategies

Two methods are possible in cartilage and bone engineering: (1) in situ tissue engineering, which involves an incorporation of an acellular scaffold matrix attracting local cells (cell homing) guiding the process of regeneration; (2) ex vivo cell seeding on the scaffold, which provides enough competent cells to orchestrate the regenerative mechanism [57]. The second strategy appears better for TMJ regeneration because of its limited capacities of self-repair and the rapid regeneration expected. Whatever the cell origin, low-intensity pulsing ultrasound on mandibular condyle enhances its regeneration [58]. In the same way, culture in spinner flasks, increases matrix production of TMJ disc cells as compared to static conditions [59].

Autogenic cells are the ideal cell source for tissue regeneration. Fibrochondrocytes from mandibular condyle seeded on polyglycolic acid (PGA) scaffolds showed weaker regenerative capacities than chondrocytes from ankle joint. Notably, they produced less GAGS and collagens [44]. In the same way, TMJ disc cells as compared to costal chondrocytes have inferior biochemical qualities and so produce less GAGs and collagens [60,61,62]. These limited capacities of TMJ fibrochondrocytes and the fact that it would be very difficult to have enough competent cells from the diseased TMJ, lead to find another sources of competent cells [63].

To regenerate TMJ condylar cartilage, primary costal-chondrocytes or hyaline cartilage cells from all cartilages in the body can be used [44,62]. Stem cells from the synovial capsule surrounding the joint can be extracted to generate new cartilage but their properties are reduced compared with other stem cells [64]. Human umbilical cord-derived mesenchymal-like stem cells (HUCM) are also proposed for TMJ regeneration [63]. Compared with the fibrochondrocytes from mandibular condyle, they promoted higher collagen types I and II, GAGs and cell colonization inside PGA scaffolds [63].

Bone marrow mesenchymal stem cells (BMSCs) provide a high rate of cell growth and division. Their advantage is the important volume of cells available and the numerous kind of possible differentiation. They can promote bone and cartilage regeneration of TMJ. Their disadvantage is their tendency to endochondral ossification [64,65,66,67].

Adipose stem cells (ADSCs) could be a potential cell source for TMJ engineering [65]. They are pluripotent mesenchymal stem cells that present multilineage differentiation [68]. These stem cells reaped from adipose tissue are easily obtainable whatever the quantity needed [67]. The implantation site of TMJ having a low vascularization, the capacity of ADSCs to undergo a low oxygen environment is very interesting. They can replicate the extracellular matrix environment of the implantation site, with the different types of collagen [65].

Different tooth-derived stem cells are also potential competent cells for TMJ regeneration. periodontal ligament stem cells (PDLSCs) and stem cells from apical papilla (SCAPs) similar to mesenchymal stem cells (MSCs) [69,70] are able to differentiate into chondrocytes and osteoblasts [71,72]. Dental follicle progenitor cells (DFPCs) which are stem cells from dental follicles involved in early tooth formation phases [73] can also differentiate into chondrocytes and osteoblasts [69,74].

Dental pulp stem cells (DPSCs) mesenchymal stem cells from dental pulp [75] are known to differentiate into different kinds of cells, such as osteoblasts and chondrogenic cells [69]. They are particularly adequate for regeneration of mineralized tissue [76]. Their multipotency, proliferation rate and availability appear better than those of BMSCs.

The capacity of osteogenic differentiation of DPSCs is well-documented [77,78,79]. DPSCs and collagen sponges showed excellent results inside human mandibular defects [71]. In a rabbit model of alveolar bone defects, it has shown high expression of BMP-2 by DPSCs as well as a high amount of bone formation [80]. This high expression of BMP-2 is the key for the differentiation of DPSCs [81,82,83]. The simple immobilization of DPSCs in scaffolds activates their osteogenic differentiation [84].

For regeneration of discal fibrocartilage, dermal fibroblasts are promising. Easily available, these autologous cells seeded in quantity and treated with IGF-1 showed a high chondrogenic potential [85]. For disc regeneration, seeding density must be carefully controlled in order to not decrease biomechanical properties. Increasing the cell number up to 1.2 × 10^8^ cells/mL of scaffold volume enhanced fibrocartilaginous deposition but modified mechanical properties. The lowest seeding density that promotes functional properties close to in vivo conditions must be identified for each cell source to regenerate TMJ disc [85].

These stem cells able to undergo both chondrogenic and osteogenic differentiation are crucial for TMJ regeneration. The best strategy should be to use a unique stem cell type able to support in a unique scaffold biphasic osteochondral regeneration orchestrated by active pro-chondrogenic and pro-osteogenic molecules.

### 4.2. Scaffolds for TMJ Cartilage Regeneration

Hyaluronic acid (HA) is a polysaccharide abundant in cartilaginous matrices, which constitutes an ideal chondrogenic microenvironment, ideal for cartilage regeneration [86]. HA hydrogels promote the differentiation of stem cells into chondrocytes and their synthesis of cartilaginous matrix [87] and support a level of chondrogenic protein expression required for cartilage regeneration [88]. Incorporation of other molecules improves mechanical properties of HA scaffolds to support cartilage tissue regeneration.

Agarose is a polysaccharide extracted from seaweed, used as agar for cell culture. Its advantage is its adaptable stiffness, which allows an easy variation of mechanical features of the scaffold [89]. Agarose scaffolds promote differentiation of different stem cells, such as MSCs and ADSCs into chondrocytes [90,91,92].

Poly-vinyl alcohol (PVA) is a hydrophilic polymer which is also very appropriate for cartilage regeneration due to its high water content and its elastic properties [93]. Its capacity to promote repair of articular cartilage is well-documented [94,95,96]. Modifications of parameters in PVA hydrogel synthesis allow suitable tensile strength [97] and elastic modulus [98] to sustain cartilage regeneration. PVA scaffolds retain long enough their chondrogenic and mechanical properties in vivo. Indeed, the rate of degradation of PVA is enough low enough to give time for cartilage to regenerate [99].

Poly-l-lactic-coglycolic acid (PLGA) is a synthetic polymer approved by the FDA for clinical applications which is greatly interesting for cartilage regeneration. The versatility of its structure allows also a modulation of mechanical properties of the scaffold. PLGA scaffolds promote colonization and differentiation of MSCs in vivo [100]. PLGA interacts positively with chondrocytes and other resident cells of the TMJ disc to regenerate. Nevertheless, it does not interact well with native collagens of the TMJ disc [101]. Incorporation of other polymers in PLGA scaffolds improves theirs chondrogenesis capacity and reduces the process of degeneration [102].

### 4.3. Scaffold for Fibrocartilage Regeneration

For the specific regeneration of TMJ disc, a variety of scaffolds have shown their efficacy in vitro and in vivo [103,104]. An aporous scaffold of polyglycerol sebacate (PGS), an elastomer, was used for regeneration of the TMJ disc. PGS scaffolds revealed to be favorable for culture of goat fibrochondrocytes and therefore for TMJ disc regeneration [105]. Poly-glycolique acid (PGA) is a biodegradable polyester. An engineered disc was proposed, made by PGA mesh scaffold-included cells [106]. Scaffolds of PGA have shown their capacity to support the culture of stem cells from human umbilical cord, their chondrogenic differentiation and expansion [63]. Poly-l-Lactic acid (PLLA) is interesting for its slow degradation rate. PLLA scaffolds seeded with porcine TMJ cells and treated with TGF β-1 improved mechanicals properties and showed higher collagen and GAGS deposition as compared to PGA scaffolds [4,107]. A mixed scaffold made by polytetrafluorethylene monofilaments, PLA monofilaments, polyamide monofilaments, and natural bone has been shown to support human and porcine disc cells culture and expansion [108]. An acellular regenerative template for reconstruction of TMJ disc made of porcine-derived extracellular matrix was studied. Implantation of this scaffold after six months showed attractive results [109].

### 4.4. Scaffold for Osteochondral Regeneration

Collagens are natural polymers very convenient for osteochondral regeneration and also for total TMJ disc reconstruction [110]. Collagens can be used as a gel which gives the opportunity to be injected into the narrow space of TMJ. Rigidity must be weak enough to allow intra-articular injection and important enough to allow cell adhesion and proliferation. Composite scaffolds incorporating collagens optimize the mechanical properties of osteochondral regenerative implants [111]. A collagen scaffold associated with GAGs increased chondrogenic differentiation of mesenchymal stem cells in a rat model [112]. Collagen scaffolds with hydroxyapatite and platelet-rich plasma promoted regeneration of entire TMJ condyles in children and adolescents suffering of TMJ ankyloses. Other clinical investigations are required to evidence the long-term efficiency of these scaffolds [113].

Gelatin, derived from the lysis of collagen is also appropriate for osteochondral regeneration. Gelatin extracellular environment is favorable to the adhesion and colonization of chondrocytes [114]. Gelatin scaffolds with chitosan have shown their capacity to support chondrogenic differentiation in vitro and in vivo [115,116].

**Figure 5:**
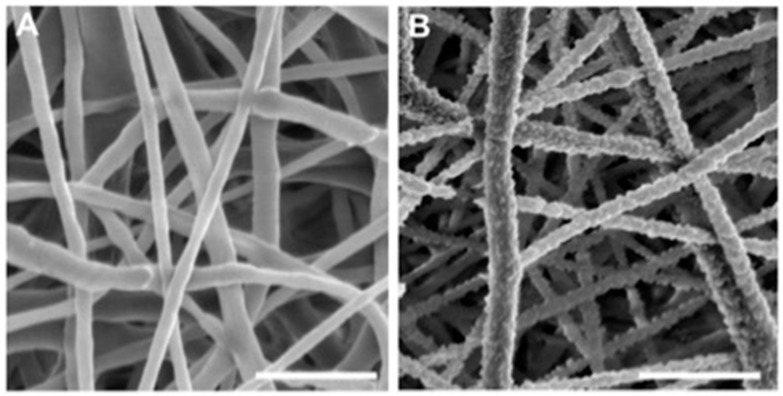
Scanning electron microscopy (SEM) observations of nanofibrous pro-regenerative biomimetic implants: Poly(ε-caprolactone) implant with an electrospun nanofiber network mimicking the pattern of the connective tissue matrix (**A**); Poly(ε-caprolactone) implant functionalized with nanoreservoirs of growth factors on the surface of nanofibers (**B**). Scale bar: 3 µm.

Nanofibers constitute pro-regenerative biomimetic extracellular matrices very interesting for tissue regeneration. The electrospinning technique makes it possible to obtain different matrices made of synthetic and natural polymers whose nanofiber diameter is close to the size of the collagen nanofibers (50–500 nm). The network of electrospun nanofibers as well as the micropores formed (less than 100 μm in diameter) mimics the structure of the connective tissue matrix [117,118]. Poly(ε-caprolactone) (PCL) is a biodegradable synthetic polymer, approved by the FDA for clinical applications. Electrospun matrices of PCL show favorable results for osteochondral regeneration [119,120,121] (Figure 5).

Fibrin presents a great interest for osteochondral regeneration. Most of studies deal with fibrin scaffolds for culture and differentiation of stem cells [89]. Fibrin-based scaffolds functionalized with adequate active molecules sustain differentiation of mesenchymal stem cells for cartilage [122,123] or bone [124,125] regeneration.

These scaffolds, able to support both cartilage and bone regeneration are crucial for TMJ regeneration. They give the opportunity to build an osteochondral construct in the same scaffold, i.e., a sole scaffold but biphasic due to its functionalization.

### 4.5. Growth Factors of Interest

Growth factors help tissue regeneration at different levels. They can promote the differentiation and proliferation of cells. They can support extracellular matrix synthesis and its mineralization [126]. They can also biologically modulate the regeneration in order to be self-limited and prevent ossification and fibrous adhesion [37].

The three key growth factors for TMJ regeneration are basic fibroblast growth factor (bFGF), insulin-like growth factor 1 (IGF-1) and transforming growth factor-β1 (TGF-β1). They are able to maintain disc-like tissue in culture [127,128] and to induce BMSCs differentiation into fibroblast-like cells, synthesizing discal matrix of type I collagen and glycosaminoglycans (GAGS) [129,130].

The effect of IGF-1 on stimulating chondrogenesis is well-documented in vitro and in vivo. In particular, it increases both GAG and collagen contents of the engineered cartilage [131,132]. The fibrochondrocytes from mandibular condyle are less responsive to IGF-1 than hyaline chondrocytes [44].

TGF-β1 shows some positive effects on cellular proliferation and on the production of extracellular matrix in TMJ disc implants [133]. It induces a significant increase in the total fraction of collagen and matrix deposition inside the engineered cartilage [134]. TGF-β1 and IGF-1 promote cellular proliferation and secretion of type I collagen and GAGs in vitro on engineered mandibular condyle [130]. TGF-β1 increased collagen synthesis, Young’s modulus and compressive stiffness in co-culture of articular chondrocytes and fibrochondrocytes [133].

The stimulating effect of bFGF on cell proliferation and production of collagen is well-known. GAGS synthesis is also significantly stimulated by bFGF [7]. bFGF and IGF-1 synergistically better promote the proliferation of disc cells [106] than the synthesis of the TMJ disc matrix [65]. In 2D culture, 10 ng/mL of bFGF increased the proliferation of fibrochondrocytes from mandibular condyle more than the 10 ng/mL of TGF-β1 and IGF-1 [135].

Platelet derivative growth factor (PDGF) significantly increases the proliferation rate of the TMJ-disc derived cells, collagen and hyaluronic acid synthesis in engineered TMJ disc. It upregulates RNA levels of type I and II collagens, matrix metalloproteinases (MMPs), and their specific tissue inhibitors (TIMPs) [136]. PDGF also significantly increases GAGs synthesis [7].

Over-expression of some interesting growth factors for tissue regeneration has been evidenced in malignant tumors. The debate about their oncogenic capability still hounds their clinical employment for tissue regeneration of the oral and maxillofacial region [137]. Finally, it is the drug delivery system of the active molecule, which is crucial.

## 5. Drug Delivery Systems

Various technologies for incorporation of growth factors into scaffolds are possible. The release of growth factors must match the rate of healing and regeneration [138,139]. The best drug delivery system can be achieved by incorporating active molecules into the scaffold. Immersion of scaffold in a solution of growth factors allows a snappy release in random distribution. Covalent binding of growth factors to the scaffold improves the control of the release. The covalent linkage may be sluggish and allows a more suitable release corresponding to cellular requests [140].

Functionalization can otherwise be accomplished by gene therapy. Gene transfer can also be conducted by viral or non-viral transduction. For tissue regeneration, the most appropriate method for gene transfer uses retroviruses, adenoviruses or adeno-associated viruses [141,142,143]. These functionalizations are optimized through nanotechnologies. Nanotechnologies could meet the challenge of the regeneration of ATM. To build drug delivery systems at a nanoscale level increases the quality of targeting and the control of distribution of the active molecules. It allows reduction of their quantity, thereby their side effects and their cost. Concentration of different active molecules allowed by nanotechnologies is also very advantageous for orchestration of different stages of TMJ regeneration and for synergetic action of growth factors.

Nanofunctionalization of scaffolds made of electrospun nanofibers is possible by different techniques: plasma or wet chemical treatment, surface graft polymerization and co-axial electrospinning [144]. The co-axial technique consists of incorporating active molecules into the polymer solution to be electrospun and so of encapsulating them inside the nanofibers for a delayed action [145]. Electrospinning can be associated with electrospraying in order to functionalize nanofibers during their production [146]. The strategy of nanofibers functionalization by BMP-2 or BMP-7 nanoreservoirs is very effective for bone regeneration. This strategy also allows the differentiation of MSCs, and accelerates the tissue regeneration in vivo [147,148,149]. Besides, co-functionalization allowed by nanoreservoirs on nanofibers can promote regeneration but also normalization of inflammation at the implantation site [150] (Figure 5B).

Intra-articular drug delivery methods applied to the TMJ seem very attractive for both pain management and regenerative strategies. Benefits of current methods of intra-articular injection are controversial. Some alarming reports describing post-injection complications have discouraged their use for TMJ pain [151,152]. The risk of complications is correlated to the number of injections and so reduced by increasing the half-life of the drug and by promoting slow-release of intra-articular medications [151,152]. Hydrogels, polymeric microparticles and liposomes are suitable drug delivery systems. They limit rapid degradation and clearance of injected active molecules and therefore avoid frequent injections and high concentrations [153]. Intra-articular drug delivery can be convenient to modify the joint environment prior to implantation or to deliver pro-regenerative molecules in a surgically controlled fashion. It avoids systemic drug release, ectopic effects and other complications [153]. Microparticles of PLGA have been revealed to be biocompatible and suitable for intra-articular delivery to TMJ in rat and therefore can support regenerative strategies [154]. Controlled release of anti-inflammatory siRNA from biodegradable microparticles of PLGA have been proposed for intra-articular delivery to TMJ [155].

## 6. Osteochondral Regeneration

Bone and cartilage regeneration occur in very different competing conditions. To engineer a biphasic osteochondral implant is therefore challenging. Ideal approaches for TMJ regeneration are a single scaffold functionalized by an osteochondral molecular gradient and a unique stem cell population associated using rapid and synchronized tissue engineering techniques [156]. Understanding molecular interactions between cells of the osteochondral interface is crucial for engineering innovative osteochondral implants [157].

In large osteochondral defects of goat condyles, PLGA composite implants seeded with Nel-related protein 1 (NRP1) modified-autologous BMSCs were able to regenerate bone and cartilage tissue after transplantation. The fibrocartilage was regenerated six weeks after transplantation and the subchondral bone native articular cartilage after 24 weeks [158].

Promising results in mandibular condyle tissue regeneration were obtained after subcutaneous implantation of athymic mice with PGA and PLA scaffolds seeded with calf osteoblasts and chondrocytes in athymic mice. Analysis after 12 weeks of implantation evidenced the condylar shape of the neoformed bone and the formation of hyaline cartilage on the articular surface and of trabecular bone [159].

Hyper-hydrated collagen gels seeded with MSCs preconditioned in two different media were proposed with one osteogenic and one chondrogenic medium at each extremity. After seven days of in vitro culture, distinct bone-like and cartilage-like areas were observed which resembled to primordial joint-like structure [156].

The same strategy of gradient-based scaffolding was proposed with PLGA microspheres: implants were functionalized with TGF-β1 at the cartilaginous end and BMP-2 at the bony end. It promoted neoformed osteochondral tissue after six weeks of implantation in mandibular condyle defects of New Zealand rabbits [158,160].

A hybrid compartmented implant was proposed with cartilage-promoting alginate/HA hydrogel at the cartilaginous end and bone-promoting nanofibrous collagen membrane at the bony end. This biphasic scaffold promoted in vitro osteogenic and chondrogenic differentiation of a single stem cell population (human MSCs). A gradient of mineralization for articular cartilage and a natural ‘glue’ at the osteochondral interface were obtained in vitro [161].

## 7. 3D Regeneration of TMJ

Whatever the strategies used, the regeneration of TMJ must match the anatomic, structural, and functional particularities of the mandibular condyle and its disc.

A bone implant of biodegradable PLGA seeded with porcine bone marrow MSCs was designed as mandibular condyle [162]. Similarly, implants of TMJ disc were engineered in the shape of TMJ discs [58,163]. Rat MSCs seeded into condyle-shaped PEG hydrogel were able to differentiate into chondrogenic and osteogenic cells [164]. Porcine derived extracellular matrix scaffolds were designed to mimic the size and shape of the TMJ. Their implantation in a canine model of TMJ discectomy led to regeneration of a functional TMJ disc [109].

The application of static uniaxial load on shape-specific TMJ disc engineered by co-culture of articular chondrocytes and meniscal fibrochondrocytes increased its functional properties. It optimized GAG synthesis and anisotropic properties resembling those of a TMJ disc [165].

Current TMJ replacement is made by prostheses. These alloplastic strategies are constantly improving in order to obtain personalized 3D prosthesis. Personalized prostheses of TMJ fabricated by 3D-printing were designed and implanted in patients. Compared with stock devices, these personalized 3D prostheses present better biomechanical and clinical outcomes. This 3D-printing technique also improves the surgery. Indeed, the positioning of an implant is easier, due to its optimal shape and to the opportunity to have an optimal 3D surgical guide [166,167,168,169,170].

These crucial clinical advances of personalized 3D prosthesis benefit regenerative strategies. Indeed, personalized 3D scaffolds can be considered. In that direction, the computer-designed nanofibrous and microporous scaffolds proposed by Chen et al. are very attractive and lead the way of a personalized 3D bone regenerative nanomedicine [171].

Currently, different techniques exist to produce 3D scaffolds such as phase separation, self-assembly, electrospinning and bioprinting. As seen previously, two points are necessary for the 3D scaffold to help and favor tissue regeneration: growth factors and living cells. Electrospinning can combine these three parameters. It allows 3D and porous structures constituted of nanofibers mimicking extracellular matrix. It can also be tuned to modulate biodegradability and resistance depending of the type of tissue to regenerate. Living cells can be added on the 3D scaffold for colonization of the matrix, or directly be included inside fibers using coaxial techniques [172,173]. 3D bioprinting also brings together the three parameters necessary for tissue regeneration. Compared to electrospinning, 3D bioprinting can reproduce structure and shape of tissues identical to those found in vivo [174]. This technique works in a layer-by-layer fashion, in which cells and growth factors can be included, allowing the control of the entire architecture of the tissues to be reproduced. These technologies participate to significant advances in tissue engineering and are promising for future clinical regenerative strategies.

A personalized 3D polyamide implant coated by nanoscale hydroxyapatite was rapidly designed and manufactured by computer in replacement of mandibular condyle. Its implantation into a patient showed positive clinical outcomes [175].

3D printed scaffolds were engineered with a spatiotemporal delivery of connective tissue growth factor (CTGF) and TGF-β3 encapsulated in microparticles in order to build a rabbit TMJ disc. Their implantation evidenced positive results. Significant improvement of regeneration was observed with the spatiotemporal gradient of functionalization [176]. Same approaches were developed to engineer human 3D-printed TMJ discs. 3D-printed scaffolds mimicking anisotropic collagen alignment of the human TMJ disc were functionalized by microparticles of CTGF and TGF-β3 and then colonized by human MSCs over six weeks. Synthesis and remodeling of the matrix promoted by this 3D growth factor delivery system allow the obtainment of an implant with heterogeneous fibrocartilaginous matrix close to a human TMJ disc. This 3D reproduction of matrix heterogeneity gives to the implant viscoelastic properties which are region-dependent and so crucial for its function in future clinical applications [177].

## 8. Conclusions

Prevalence of affections of TMJ is important. Severe affections are preferentially concerned by regeneration. Currently, they are treated by arthrotomy and implantation of prostheses. The recent advances in regenerative medicine for orthopedics may provide solutions for TMJ regeneration. However, anatomic, structural, and functional regeneration of TMJ is very challenging and specific. The fibrocartilaginous property of the mandibular condyle and its tight link with its fibrocartilaginous disk contribute to modifying issues. The difficulty is not to obtain a pure hyaline cartilage with an underlying bone as for the other articulations. The main issue is to get a long-term fibrocartilage well-separated from its underlying bone without ossifications or fibrous adhesions which are dramatic for crucial oral functions of patients. At present, concrete progress of TMJ arthroscopy allows adequate visualization and manipulation of pathological intra-articular tissues and motivate the emergence of innovative and specific regenerative strategies of TMJ. Numerous proposals of interest have been presented focusing on suitable cells, scaffolds or active molecules for TMJ regeneration. Global strategies, able to support the entire mandibular condyle regeneration, are very attractive. So, the desired approach is a unique scaffold inducing an osteochondral molecular gradient containing a single stem cell population able to undergo osteogenic and chondrogenic differentiation such as BMSCs, ADSCs or DPSCs. The key to this complex regeneration is the functionalization by active molecules such as IGF-1, TGF-β1 or b-FGF. This regeneration can be optimized by nano/micro-assisted functionalization and by spatiotemporal drug delivery systems orchestrating the 3D formation of TMJ tissues.
